# Improving Mobility: A Case Report on the Rehabilitation of a Gait Anomaly in an Asian Elephant at a Thai Elephant Conservation Center

**DOI:** 10.3390/ani15111632

**Published:** 2025-06-02

**Authors:** Siriphan Kongsawasdi, Kittichai Wantanajittikul, Therdchai Jivacate, Warangkhana Langkaphin, Saran Chansitthiwet, Petthisak Sombutputorn, Kittikul Namwongprom, Narueporn Kittisirikul, Siripat Khammesri, Taweepoke Angkawanish

**Affiliations:** 1 Faculty of Associated Medical Sciences, Chiang Mai University, Chiang Mai 50200, Thailand; siriphan.k@cmu.ac.th (S.K.);; 2 Elephant, Wildlife and Companion Animals Research Group, Chiang Mai University, Chiang Mai 50100, Thailand; 3 Faculty of Medicine, Chiang Mai University, Chiang Mai 50200, Thailand; 4 National Elephant Institute, Forest Industry Organization, Lampang 52190, Thailand

**Keywords:** elephant gait, rehabilitation, forelimb lameness

## Abstract

This case report details the successful rehabilitation of a 31-year-old male Asian elephant (*Elephas maximus*) presenting with an abnormal left forelimb gait following chronic traumatic injury. The elephant exhibited a distinctive circumduction gait with a semicircular arc movement, characterized by limited flexion at the elbow and carpus, along with compensatory proximal shrugging during the swing phase. Diagnostic evaluations revealed joint space narrowing and ligament fibrosis, while biomechanical gait analysis using inertial measurement units highlighted significant asymmetries between affected and unaffected limbs. An interprofessional team developed a comprehensive rehabilitation protocol that integrated peripheral magnetic stimulation, task-specific therapeutic walking with adjustable obstacles, and progressive strengthening exercises. At the eight-week follow-up, improvements were observed in cross-correlation coefficients of limb movement and imaging assessments, indicating enhanced symmetry and structural improvements with reduced fibrosis. However, persistent discrepancies in elbow functions suggest that further targeted rehabilitation may be warranted. This report underscores the potential of a coordinated interprofessional approach to restore functional gait patterns in elephants and offers valuable insights for future rehabilitative strategies in managing complex musculoskeletal injuries in large mammals.

## Simple Summary

This case study details the successful rehabilitation of a 31-year-old male Asian elephant (*Elephas maximus*) with a chronic injury to its left forelimb. The elephant exhibited circumduction gait, moving its left shoulder in a semicircular arc to clear the ground. Diagnostic evaluations revealed chronic inflammation of the medial collateral ligament in the elbow, and gait analysis of the lame limb demonstrated major asymmetries compared to the contralateral limbs. Multidisciplinary professionals developed a comprehensive rehabilitation protocol incorporating peripheral magnetic stimulation and strengthening exercises with therapeutic walking. After ten weeks, limb symmetry improved, and some fibrotic abnormalities disappeared. This case highlights how a multidisciplinary approach can facilitate restoration of normal walking patterns in elephants, offering suggestions and strategies for treating complex musculoskeletal ailments in large mammals.

## 1. Introduction

Elephants exhibit distinctive locomotion and gait characteristics due to their large body size and unique anatomical structure. They use a lateral sequence gait in which the hindfoot follows the forefoot on the same side—a pattern maintained across all speeds [1]. Anatomical adaptations and biomechanical features include a unique distribution of foot pressure, supported by a specialized fat pad and a distinctive false toe (the prepollex in the forelimb and prehallux in the hindlimb) at the heel [2,3]. Consequently, their unique locomotion pattern has evolved as an energy-efficient solution for supporting their immense body weight while preserving mobility [1,2,3].

Musculoskeletal pathologies in elephants, particularly those kept in captivity, are common and often associated with degenerative joint diseases (DJD), including osteoarthritis and foot lesions such as pododermatitis, cracked nails, and sole abscesses, which may progress to connective tissue disorders and conformational abnormalities [4,5]. These musculoskeletal problems present unique challenges in veterinary medicine as they can arise from a variety of factors, resulting in locomotor dysfunction. These conditions profoundly impact an elephant’s overall quality of life, social interactions, and ability to exhibit natural behaviors. Treatment generally involves analgesia with NSAIDs and opioids, alongside topical treatments such as Mohs’ paste (zinc chloride), copper naphthenate, and iodine foot soaks to address infections and promote wound healing [6]. Despite the critical role of mobility in elephant health and welfare, rehabilitation protocols for managing gait abnormalities in elephants remain scarce in the veterinary literature.

This case report describes the rehabilitation of an adult Asian elephant (*Elephas maximus*) presenting with an abnormal gait pattern due to chronic traumatic injury. Treatment was complicated by the elephant’s unique anatomical and biomechanical characteristics, including unusual weight distribution, specialized foot structures, and the need to support their substantial body mass.

This case is significant not only for its successful outcome but also for its novel adaptation of established rehabilitation principles for elephant care. The protocol integrates traditional husbandry practices with modern rehabilitation techniques, offering practical insights for veterinarians managing similar cases.

## 2. Detailed Case Description

**Figure 1:**
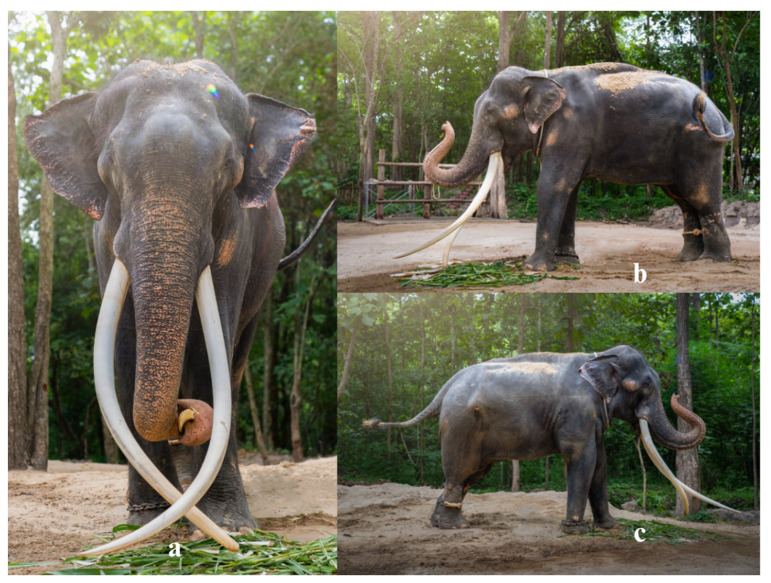
(**a**) Front view, (**b**) left view, and (**c**) right view during the stance of an elephant with a characteristic semicircular arc movement of the left forelimb. This altered motion pattern directly affects weight bearing on the affected limb. The image was taken during the initial week of the elephant hospital’s referral.

**Figure 2:**
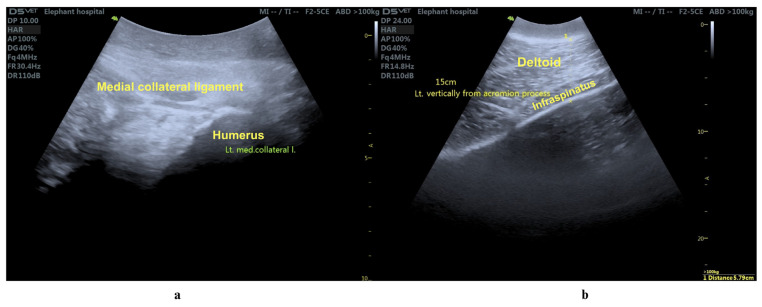
Ultrasonographic evaluation of the left medial collateral ligament (**a**) and infraspinatus muscle (**b**). Chronic fibrotic changes (arrows) and hyperechoic lesions (arrowheads) are evident in the medial collateral ligament, while the infraspinatus muscle shows significant thinning, suggesting pronounced muscle atrophy.

A 31-year-old male elephant weighing 4019 kg, 2.64 m shoulder height and a body condition score of 3.5 on a 5-point scale (normal to slightly overweight) [7] was referred to the Thai Elephant Conservation Center (TECC) hospital in September 2023 for evaluation of an abnormal left forelimb gait. After being officially relocated from Thailand to another country, the elephant lived at a prominent temple from age 8 to 30. Over time, it developed a chronic musculoskeletal condition of unknown origin resulting in significant discomfort and changes to its gait pattern. On examination, the left forelimb exhibited a semicircular arc movement, remaining straight from shoulder to foot with motion originating at the shoulder joint, while both the elbow and carpus remained extended throughout (Figure 1). A multidisciplinary care team comprising veterinarians from TECC and the Faculty of Veterinary Medicine, Chiang Mai University (CMU) as well as physiotherapists from the Faculty of Associated Medical Sciences, CMU, performed the physical examination and laboratory investigation. Ultrasonographic imaging revealed chronic fibrotic changes in the left medial collateral ligament (arrows, Figure 2a) and hyperechoic lesions in the triceps tendon (arrowheads, Figure 2b), alongside significant thinning of the left infraspinatus muscle (Figure 2b). Pronounced atrophy of the infraspinatus muscle was also observed. Chronic fibrotic changes in the medial collateral ligament and significant thinning of the infraspinatus muscle, as demonstrated in the ultrasonographic evaluation, indicate advanced degeneration of the upper portion of forelimb’s musculotendinous structure. The infraspinatus, a component of the rotator cuff complex, plays a crucial role in propulsion, gravitational support, and stabilization in megaherbivores [8]. Follow-up ophthalmic examination revealed bilateral lens luxation, and ocular ultrasonography confirmed a cataract in the right eye. However, further assessments indicated that the cataract did not impair the elephant’s vision or affect its gait. Gait analysis was performed using wearable sensors containing inertial measurement units (IMUs) to capture movement parameters in three-dimensional space [9,10].

### Rehabilitation Regimens

The medical team concluded that the elephant exhibited chronic inflammation of the collateral ligament in the left elbow, resulting in limited joint movement and proximal compensatory shoulder elevation in a semicircular arc to clear the floor during the swing phase of gait. Consequently, a multidisciplinary collaboration among orthopedic, veterinary, and physiotherapy specialists developed a comprehensive rehabilitation protocol with the primary goal of restoring normal gait patterns to the best extent possible.

The rehabilitation approach focused on reducing chronic inflammation associated with ligament fibrosis and implementing a strengthening program to improve forelimb muscle strength. To mitigate chronic inflammation, peripheral magnetic stimulation (PMS) up to 2.5 Tesla (BTL-6000 SUPER INDUCTIVE SYSTEM ELITE, BTL Medical Technologies Ltd., Prague, Czech Republic) was applied to the fibrous tissue surrounding the medial collateral ligament. PMS at lower intensities (0.375 to 0.750 Tesla) was also applied to stimulate muscle activity in atrophic muscles, with the specific settings adjusted based on muscle response and elephant’s behavior.

**Figure 3:**
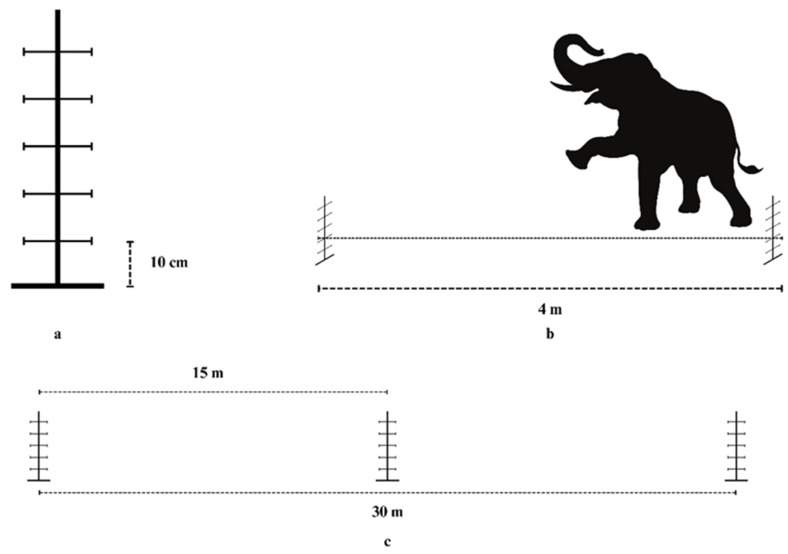
Illustration of therapeutic walking with obstacles. The elephant walked along a 30 m open courtyard with a hard ground surface, navigating three obstacles placed at 15 m intervals. Each obstacle (**a**) consisted of adjustable horizontal poles with heights ranging from 10 cm to 50 cm, spaced 4 m apart (**b**). Three such obstacles were included per round of exercise (**c**). The program focused on promoting linear left forelimb movement without shoulder circumduction.

**Figure 4:**
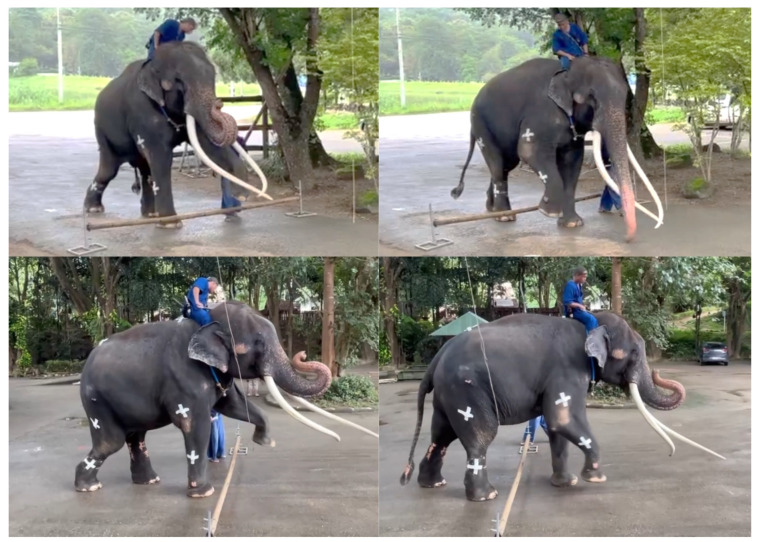
Therapeutic walking with obstacles to encourage linear left forelimb movement patterns.

To complement the reduction in fibrosis, a strengthening exercise program was implemented. This included therapeutic walking along a 30 m open courtyard on a hard ground floor, with an emphasis on guided, task-specific exercises designed to promote linear movement of the left forelimb and eliminate shoulder circumduction. Obstacles were placed at 15 m intervals (Figure 3 and Figure 4) with adjustable heights ranging from 10 cm to 50 cm. This program was carefully supervised and regulated by a mahout. When the elephant was able to lift its left forelimb in flexion of the shoulder, elbow, and carpus without using compensatory patterns, obstacle height was increased in 10 cm increments.

This exercise regimen was performed for 30 min daily, six days a week, with progressive adjustments aimed at maximizing recovery and minimizing compensatory movement patterns that could exacerbate symptoms. All treatments were delivered each morning, five days per week, from June 2024 to February 2025. Regular nail and toe care were also essential components of the elephant’s recovery. Following the musth period, thorough nail trimming was performed every 3–4 months to correct shape-related deformities that could otherwise disrupt proper weight distribution.

## 3. Results

### 3.1. Clinical Evaluation

**Figure 5:**
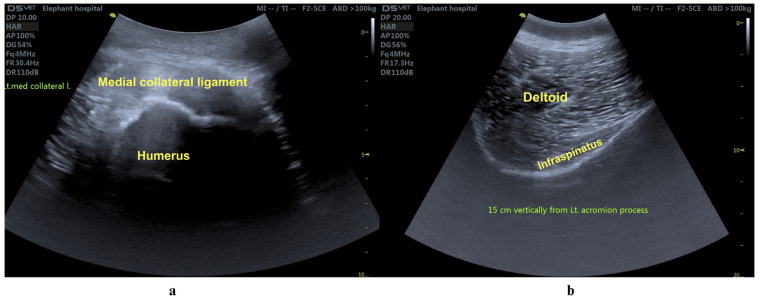
Ultrasonographic evaluation of the left medial collateral ligament (**a**) and infraspinatus muscle (**b**). Structural improvements include reduced fibrosis (arrows) and enhanced ligament echogenicity (**a**), correlating with partial reversal of the left infraspinatus muscle atrophy (**b**) by early 2025.

At initial admission in July 2024, the elephant weighed 4200 kg and had a body condition score of 4, indicating satisfactory general health. Repeated ultrasonographic assessments from September to December 2024 showed gradual improvement in tendon texture and reduced hyperechoic areas, suggesting partial decreases in fibrosis. By early 2025, structural enhancements were observed, including reduced fibrosis (arrows, Figure 5a) and increased echogenicity in the medial collateral ligament, correlating with partial reversal of left infraspinatus muscle atrophy (Figure 5b).

### 3.2. Biomechanical Gait Analysis

**Figure 6:**
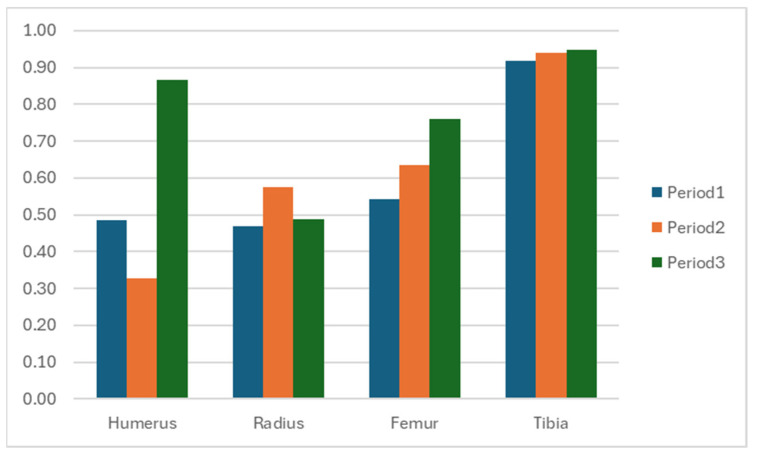
Graphical representation of cross-correlation coefficients across 3 periods.

**Figure 7:**
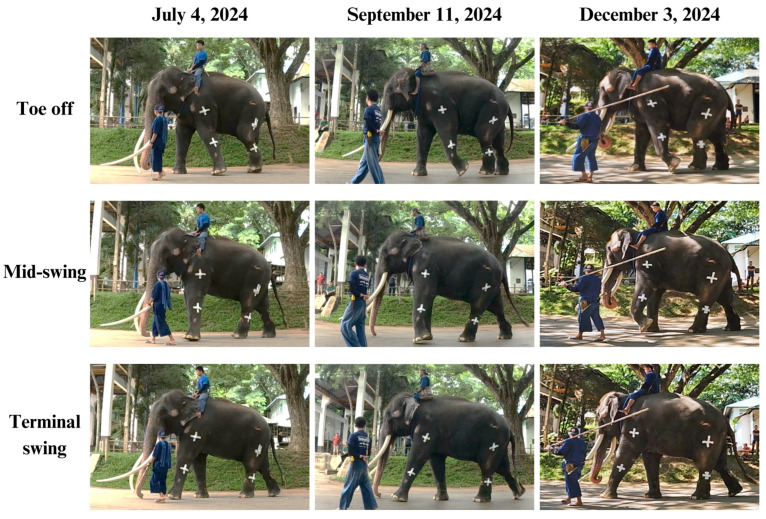
Comparison of swing phase components from initial (toe-off) to mid-swing and terminal swing across the 3 periods.

Vertical movement data derived from IMUs reflects the rhythmic vertical displacement of the body’s center of mass during gait and locomotion [11,12,13]. In the sagittal plane, this displacement typically follows a sinusoidal pattern during the normal gait cycle. This rhythmic movement is essential for maintaining balance and forward momentum during walking. In studies of animal lameness, the withers exhibit reduced vertical displacement during lame diagonal stance compared to contralateral diagonal stance [14,15]. In our study, we analyzed cross-correlation coefficients of vertical displacement between contralateral forelimbs and hindlimbs to assess movement symmetry. These coefficients indicate changes in weight-bearing and stride mechanics [13]. Five consecutive gait cycles were selected from the middle of each session to reduce the influence of transitional movements at the start and end of walking. Data were collected via IMUs attached to both the proximal and distal segments of the forelimbs and hindlimbs. Raw vertical acceleration signals were processed using custom MATLAB software (version R2023b; Mathworks, Natick, MA, USA). The signals were filtered using a low-pass Butterworth filter to eliminate high-frequency noise. The filtered acceleration data were then double integrated to derive vertical displacement over time, and the results were converted to millimeters. To account for variability in walking speed, displacement signals were normalized to a 0–1 range before conducting cross-correlation analysis. Each gait cycle was aligned at its lowest vertical point, corresponding to the initial stance phase. Cross-correlation values closer to +1 indicate greater synchronization between left and right limbs, while lower values suggest asynchronous movements that may reflect compensatory strategies or abnormal gait patterns. Cross-correlation analysis was performed at three points: Period 1 (July 2024) following the musth period when treatment was suspended, Period 2 (September 2024), and Period 3 (December 2024), the final follow-up (Table 1 and Figure 6 and Figure 7).

During each gait cycle, the swing phase occurs when the foot lifts off the ground and the leg advances toward the next step. It begins immediately after toe-off of one foot, termed initial swing, and ends just before heel strike of the same foot, known as the terminal swing [16,17]. For a normal swing to occur, proximal joints such as the shoulder and elbow must flex to initiate the movement. This flexion continues throughout the mid-swing phase, culminating in elbow extension during terminal swing, which prepares the heel for ground contact while the shoulder remains flexed to ensure proper foot placement. These coordinated actions allow the leg to propel forward, navigate obstacles, and align appropriately for the next step [18]. However, this elephant initially exhibited an abnormal circumduction gait pattern in the left forelimb during the swing phase. This was reflected in low cross-correlation coefficients for humerus (0.49) and radius (0.47), indicating significant asymmetry between the affected and unaffected sides. The asymmetry was also detected in the proximal part of the hindlimbs (coefficient 0.54), while the distal hindlimbs did not exhibit such discrepancies. This imbalance may be due to uneven weight distribution, as the forelimbs bear approximately 60% of body weight compared to 40% by the hindlimbs. At the second follow-up in September 2024 (two months after initial assessments), improvements were observed in the symmetric indices of the radius, femur, and tibia. The radius demonstrated a modest improvement (from 0.47 to 0.58), suggesting increased elbow joint contribution to gait. Likewise, gradual improvement in the femur and tibia suggested reduced compensatory movement in the hindlimbs. In contrast, the humerus showed a decline in its coefficient (0.49 to 0.33), indicating continued circumduction. Repeated ultrasonographic assessments in December 2024 (Period 3) revealed improved tendon texture and reduced fibrosis in the medial collateral ligament. However, a new issue was identified: the elephant’s enlarged tusk disrupted normal locomotion by increasing forelimb elevation during movement. In response, the exercise rehabilitation program was subsequently modified by eliminating the obstacles and facilitating the elephant’s ambulation on level terrain in a figure of 8 pattern walk, under the supervision of its mahout and veterinarian. At the follow-up period, the elephant demonstrated smooth and coordinated movement, resulting in a significant increase in cross-correlation at the humerus (0.33 to 0.87). The hindlimbs also exhibited symmetric patterns as indicated by cross-correlation coefficients of 0.76 for the femur and 0.95 for the tibia. Nevertheless, the radius symmetry showed inconsistency (0.49), suggesting that the elbow function had not been completely restored and warranted a further careful rehabilitation plan.

**Figure 8:**
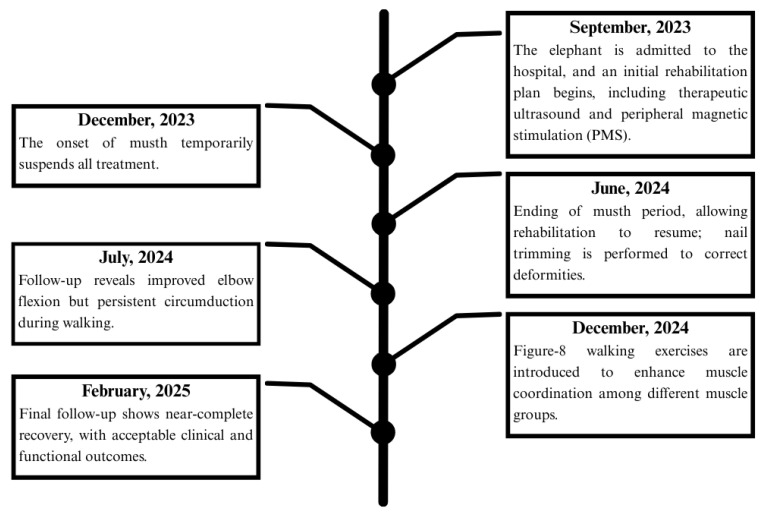
Diagrammatic representation of the rehabilitation procedure agendas.

Figure 8 illustrates the rehabilitation timeline, beginning with the elephant’s admission in September 2023 for diagnosis and initial treatment, including magnetic stimulation and therapeutic exercises. Treatment was paused during the musth period (December 2023) and resumed in June 2024, with additional interventions such as nail trimming to correct deformities. By July 2024, elbow flexion had improved, but circumduction persisted. In December 2024, the rehabilitation plan was updated with figure of 8 walking exercises. At the final follow-up in February 2025, the elephant showed near-complete recovery, and the outcome was deemed satisfactory by the veterinary team.

## 4. Discussion

Elephants demonstrate a weight distribution pattern with approximately 60% of body weight supported by the forelimbs and 40% by the hindlimbs. Though similar to other quadrupeds, they possess specialized biomechanical adaptations including columnar limbs and fatty foot pads [19] and employ a distinctive lateral sequence gait (left hind, left front, right hind, right front) that maintains stability across all speeds by ensuring continuous ground contact with at least two feet. This gait pattern features synchronized braking and propulsion between fore- and hindlimbs without functional specialization, maintaining mechanical compliance and minimizing peak forces despite their substantial body mass [19,20,21,22]. Muscular adaptations further help to distribute this weight effectively during walking. The robust structure of the shoulder joint, characterized by powerful muscles and ligaments, permits elephants to maneuver their limbs efficiently without compromising balance [20]. When animals experience pain or discomfort in their limbs, they may adopt compensatory gait patterns to reduce load on the affected areas, often resulting in abnormal locomotion patterns [14,23,24]. In this case report, a single instance involving an Asian elephant that sustained an injury of unknown origin, led to a circumduction gait, a semicircular swing of the left forelimb at the shoulder to clear the ground. This altered gait pattern induces biomechanical compensations that impose cumulative stress on other musculoskeletal structures resulting in profound atrophy of the infraspinatus muscle due to disuse, along with fibrosis of the collateral ligaments at the elbow epicondyle. This pattern may have detrimental effects; the increased weight shift to the contralateral non-lame forelimb during stance phases may cause stress on the collateral ligaments, predisposing the animal to carpal hyperextension injuries and long-term osteoarthritis. The circumduction gait shifts propulsion demands to the ipsilateral hindlimb, causing excessive torque during limb protraction and increasing sacroiliac ligament strain, which is associated with lumbo-sacral pain. Moreover, increased thoracic spine curvature over the contralateral forelimb reduces intervertebral disk space and may lead to spinal deformities such as scoliosis and kyphosis [23,24,25,26]. Therefore, the multidisciplinary health care team, comprising specialists in human orthopedic surgery, veterinary medicine, physical therapy, and biomedical engineering, collaborated to develop an appropriate rehabilitation program aimed at reducing chronic inflammation, improving muscle strength, and promoting normal gait patterns.

Several forms of magnetic therapy are employed in rehabilitation settings, including Pulsed Electromagnetic Field Therapy (PEMF), Transcranial Magnetic Stimulation (TMS), and Magnetic Resonance Therapy, each with distinctive mechanism and clinical application. Peripheral Magnetic Stimulation (PMS) is a specialized modality that targets peripheral nerves and muscles. As a non-invasive neuromodulatory technique, PMS offers targeted pain relief and supports functional recovery across various musculoskeletal and neurological conditions [27,28,29]. It activates peripheral nerves and muscles through high-intensity magnetic pulses, which modulate neural activity, enhance blood flow, and promote tissue repair. Unlike electrical stimulation techniques, which rely on direct impulses, PMS generates eddy currents that depolarize motor neurons without direct contact, inducing involuntary muscle contractions while enhancing corticospinal excitability and facilitating voluntary movements. Additionally, PMS induces more natural and coordinated muscle activation patterns due to its ability to indirectly stimulate larger areas of the motor cortex [28]. This can lead to a more synchronized recruitment of muscle units, mimicking natural movement patterns. It is generally considered more comfortable for patients as it does not involve painful electrical impulses. The non-invasive nature of PMS allows for longer application times without significant discomfort [30]. It is particularly effective in rehabilitation contexts where coordination and natural movement patterns are critical, such as post-stroke recovery or injury rehabilitation.

Task-specific exercises in animal rehabilitation refer to training methods that are designed to target specific motor tasks, thereby focusing on relearning functions that are meaningful and practical for the animal [31,32]. They provide substantial benefits in animal rehabilitation, especially for this case involving a standardized obstacle navigation protocol. This approach facilitates targeted neuromuscular retraining that directly translates to functional movement patterns required in the animal’s environment. By systematically progressing obstacle heights (10–50 cm) and maintaining consistent spacing (2 m intervals), the protocol enables precise calibration of challenge levels while the mahout’s guidance ensures proper movement execution. This methodology not only addresses the specific movement deficits but also capitalizes on motor learning principles by incorporating meaningful, practical tasks that promote neural adaptation and movement pattern normalization. Evidence in humans suggests that such functional training enhances recovery outcomes in both neurological and musculoskeletal conditions by engaging integrated sensorimotor pathways more effectively than isolated interventions [33,34,35].

Nevertheless, this study describes a single case and lacks a confirmed diagnosis of the initial injury, our understanding of the pathophysiological mechanisms underlying both the gait abnormality and recovery remains incomplete. Additionally, the relatively short follow-up period limits our ability to assess long-term outcomes and potential relapses. Despite these constraints, this case provides valuable preliminary evidence for rehabilitation approaches in elephants with similar gait anomalies. Future research should include larger sample sizes, longer follow-up periods, and more detailed diagnostic imaging to establish clearer treatment protocols for various musculoskeletal conditions affecting elephants’ mobility.

## 5. Conclusions

This case report demonstrates that a coordinated, multidisciplinary approach can effectively restore normal gait and improve the long-term quality of life in elephants suffering from complex musculoskeletal and neurological impairments. The integration of targeted interventions—such as PMS and task-specific exercise protocols—along with specialized expertise from orthopedic surgery, veterinary medicine, physical therapy, and biomedical engineering, proved instrumental in addressing the compensatory gait abnormalities observed in the affected elephant. Successful rehabilitation underscores the importance of tailored, multidisciplinary management strategies in treating large mammals with musculoskeletal conditions and highlights the potential for establishing standardized rehabilitation protocols. Future research should aim to further refine these therapeutic approaches to enhance recovery outcomes and overall animal welfare in similar cases.

## Figures and Tables

**Table 1:** Cross-correlation coefficients of vertical displacement of forelimb and hindlimb at proximal and distal parts.

Segment	Period 1 Mean (sd)	Period 2 Mean (sd)	Period 3 Mean (sd)
Forelimb; Humerus	0.49 (0.01)	0.33 (0.07)	0.87 (0.03)
Forelimb; Radius	0.47 (0.01)	0.58 (0.00)	0.49 (0.11)
Hindlimb; Femur	0.54 (0.01)	0.64 (0.01)	0.76 (0.04)
Hindlimb; Tibia	0.92 (0.01)	0.94 (0.00)	0.95 (0.01)

## Data Availability

The data analyzed for this study are available from the corresponding author upon reasonable request.
